# Vascular Endothelial Growth Factor (VEGF) isoform expression and activity in human and murine lung injury

**DOI:** 10.1186/1465-9921-10-27

**Published:** 2009-04-09

**Authors:** Andrew RL Medford, Samantha K Douglas, Sofia IH Godinho, Kay M Uppington, Lynne Armstrong, Kathleen M Gillespie, Berendine van Zyl, Terry D Tetley, Nassif BN Ibrahim, Ann B Millar

**Affiliations:** 1 Department of Clinical Science at North Bristol, University of Bristol Paul O'Gorman Lifeline Centre, Southmead Hospital, Westbury-on-Trym, Bristol, BS10 5NB, UK; 2 Lung Cell Biology, National Heart & Lung Institute, Imperial College, Dovehouse Street, London, SW3 6LY, UK; 3 Department of Pathology, North Bristol NHS Trust, Frenchay Hospital, Frenchay Park Road, Frenchay, Bristol, BS16 1LE, UK

## Abstract

**Background:**

The properties of vascular endothelial growth factor (VEGF) as a potent vascular permogen and mitogen have led to investigation of its potential role in lung injury. Alternate spliced VEGF transcript generates several isoforms with potentially differing functions. The purpose of this study was to determine VEGF isoform expression and source in normal and ARDS subjects and investigate the expression and regulation of VEGF isoforms by human alveolar type 2 (ATII) cells.

**Methods:**

VEGF protein expression was assessed immunohistochemically in archival normal and ARDS human lung tissue. VEGF isoform mRNA expression was assessed in human and murine lung tissue. Purified ATII cells were cultured with proinflammatory cytokines prior to RNA extraction/cell supernatant sampling/proliferation assay.

**Measurements and Main Results:**

VEGF was expressed on alveolar epithelium, vascular endothelium and alveolar macrophages in normal and ARDS human lung tissue. Increases in VEGF expression were detected in later ARDS in comparison to both normal subjects and early ARDS (p < 0.001). VEGF_121_, VEGF_165 _and VEGF_189 _isoform mRNA expression increased in later ARDS (p < 0.05). The ratio of soluble to cell-associated isoforms was lower in early ARDS than normal subjects and later ARDS and also in murine lung injury. ATII cells constitutionally produced VEGF_165 _and VEGF_121 _protein which was increased by LPS (p < 0.05). VEGF_165 _upregulated ATII cell proliferation (p < 0.001) that was inhibited by soluble VEGF receptor 1 (*sflt*) (p < 0.05).

**Conclusion:**

These data demonstrate that changes in VEGF isoform expression occur in ARDS which may be related to their production by and mitogenic effect on ATII cells; with potentially significant clinical consequences.

## Introduction

Functional and physical failure of the alveolar capillary membrane is a pivotal event in the development of lung injury, exemplified by the acute respiratory distress syndrome (ARDS)[1]. The characteristics of vascular endothelial growth factor (VEGF), as both an angiogenic and permogenic factor has led to interest in its potential role in this condition[2,3]. It is known that VEGF protein is compartmentalized within the lung[4] and alveolar type 2 epithelial (ATII) cells have been identified as a major source of VEGF in both animal studies and human foetal lung studies[5,6]. Observational data show plasma VEGF levels rise and intrapulmonary (ie, measurable in the epithelial lining fluid (ELF) obtained by broncho-alveolar lavage) levels fall in the early stages of lung injury with normalization of both during recovery[7,8]. These changes in intrapulmonary VEGF have been confirmed in ARDS but have also been observed in other conditions in which alveolar injury may occur, such as high-altitude pulmonary oedema [9-11]. To explore the significance of these observations, it is necessary to understand the mechanisms that regulate VEGF bioactivity.

Alternative splicing of the VEGF transcript from exons 5 to 8 leads to the generation of several different isoforms with variable diffusibilities depending on their length: VEGF_121_, VEGF_165_, VEGF_189 _being the main forms[3,12-14]. Exon 6 (not present in VEGF_121 _and VEGF_165_) and exon 7 provide heparin-binding affinity, exon 8 (present in all active isoforms) is necessary for the stimulation of mitosis[15]. The longer isoforms are highly basic and remain virtually completely cell-associated, whereas VEGF_121 _(lacking both exons 6 and 7) is freely diffusible[14]. VEGF_165 _(lacking exon 6 but not 7) possesses intermediary properties being largely soluble but a distinct fraction remains cell-associated[14]. It is the predominant isoform and most biologically active in the physiological state[15].

Recovery from lung injury/ARDS requires functional/physical repair of the alveolar epithelial surface to occur. ATII cells proliferate and differentiate into alveolar epithelial type 1 (ATI) cells to regenerate the alveolar epithelium after injury[16]. Limited and conflicting data exists on the effect of VEGF on alveolar epithelial proliferation; fetal human lung cells[17,18] and pulmonary adenocarcinoma derived cell lines proliferate[19], whereas adult rat ATII cells do not[20]. There are no similar studies of adult human lung epithelial cells.

We initially hypothesised that changes in VEGF intrapulmonary levels observed by ourselves and others would be reflected in whole lung tissue and associated with changes in VEGF isoforms in ARDS lung which differ in early and late stages of the disease. We further hypothesised that the role of VEGF in the lung may be as an epithelial mitogen integral to lung repair. We have explored this hypothesis and additionally considered the effects of known inflammatory mediators, previously suggested to be involved in the pathogenesis of ARDS, in these processes.

## Methods

### Specimens

Archival normal and ARDS lung tissue sections and paraffin blocks for which consent for research had been obtained, were utilized for immunohistochemistry (ARDS had been histopathologically confirmed and groups divided into either "early ARDS" within 48 hours or "later ARDS" after day 7). ARDS had been diagnosed according to the internationally used 1994 American-European Consensus Conference criteria[21]. Isolation of ATII cells was undertaken from macroscopically normal lung tissue sections (15 × 5 × 5 cm approximately) were donated by 13 patients (5 females and 8 males) undergoing lobar resection for malignancy. The median age was 71. Ethical approval was obtained from the North Bristol and United Bristol Healthcare Trusts.

### Immunohistochemistry for VEGF

Normal, early ARDS and later ARDS lung tissue sections were examined (n = 8 for each group). Normal lung tissue implied that there was no lung involvement in the cause of death. Paraffinised 4 μm sections were de-waxed in serial xylene (BDH Laboratory Supplies, Poole, UK), dehydrated in absolute ethanol (BDH Laboratory Supplies, Poole, UK) and pressure cooked in 0.01 M tri-sodium citrate (BDH Laboratory Supplies, Poole, UK) buffer (pH 6) to facilitate antigen retrieval. Endogenous peroxidase was blocked with 3% hydrogen peroxide (BDH Laboratory Supplies, Poole, UK) in methanol (BDH Laboratory Supplies, Poole, UK). Sections were incubated in 2.5% horse blocking serum (Vectastain Universal Quick Kit, Vector Laboratories, Peterborough, UK) prior to Avidin D and Biotin blocking sera (Vector Laboratories, Peterborough, UK). A rabbit polyclonal antibody to VEGF Autogen Bioclear, UK Ltd, Wiltshire, UK) and isotypic rabbit IgG controls (Vector Laboratories, Peterborough, UK) were used. Isotypic control antibodies were used on normal, early ARDS and later ARDS tissues. The samples were then stained with pan-specific biotinylated antibody, streptavidin-peroxidase complex with diaminobenzidine substrate (Vectastain Universal Quick Kit, Vector Laboratories, Peterborough, UK), counterstained in haematoxylin (BDH Laboratory Supplies, Poole, UK).

Image capture and semi-quantitative densitometry were achieved using Histometrix version 6 software version 1.4 (Kinetic Imaging) linked to a JVC TK-C1360B camera with a resolution of 470 TV lines. Pixels representing immunopositivity were chosen and this threshold was memorised by the software. Anything within the selected pixel range was accounted for and expressed as a percentage of the pixels in the selected area. This gave a composite intensity score per unit area derived from the staining intensity value divided by the staining cross-sectional area assessed. Densitometry was performed on all slides from the same procedure assigning the same random coloration as unit of intensity on each slide. Five randomly chosen (computer generated) areas on each section for each patient were assessed giving twenty values. Densities on negative control sections were subtracted from positively stained section densities to control for differing background pixel intensities detected.

### Formalin-fixed paraffin embedded (FFPE) RNA extraction and measurement

This method is a modified version of the Krafft technique[22]. Briefly, 8 × 6 μm sections were cut on a microtome from formalin-fixed paraffin-embedded (FFPE) blocks of archival normal, "early" (within 48 hours) and "later" ARDS (after day 7) lung tissue. Sections were de-waxed and washed in Histoclear II and absolute ethanol. Sections were then dried at 55°C for 3 minutes before being digested for 6 hours in digest buffer (1 mg/ml proteinase K, 20 mM Tris, 20 mM EDTA buffer at pH 7.4, 1% SDS) at the same temperature. After ice cooling, RNA was extracted using phenol:chloroform:isoamyl ethanol mixture before precipitation in sodium acetate, washing and pelleting.

### Animal model of lung injury

Male C57BL/6 mice (18–20 g) were used in a LPS induced model of lung injury. The experiments were done in accordance with Home Office guidelines. Briefly mice were exposed to intranasal LPS (Sigma, Poole, UK; serotype 011:B4) dissolved in pyrogen-free saline. Animals were lightly anaesthetised by placing in a vapour-filled chamber with halothane (Merial Animal Health). While anaesthetised, intranasal inoculation of 50 μl of 10 μg LPS/mouse was performed. This procedure was repeated daily for 4 days. Control animals were treated with the same volume of pyrogen-free saline. Animals were sacrificed by halothane hyperanaesthesia and exsanguinated by cardiac puncture. The lungs were removed, inflated and snap frozen at day 2 and day 5 post initial LPS insult, 24 hours after last LPS dose. A minimum of n = 4 individual specimens was utilized in each experiment. Lung injury was confirmed by histological analysis and RNA extracted as previously described[23]. Quantitative real-time PCR was undertaken in the day 5 post LPS mice only.

### Isolation and purification of ATII cells

ATII cells were purified according to the method of Witherden et al[24] as previously described. Sections were perfused with 0.9% saline, digested with 0.25% trypsin (Sigma, Poole, UK) and minced with newborn calf serum (NCS) (Invitrogen, Paisley). DNAse I was added to the suspension at 250 μg/ml in 7 ml Hanks Balanced Salt Solution (HBSS). The suspension was shaken before filtering through a ~500 μm filter and then 40 μm mesh (Fahrenheit, Milton Keynes). ATII cells were purified by differential adhesion. The non-adherent cells were centrifuged at 300 g for 10 minutes at 4°C and resuspended at 1 × 10^6 ^ATII cells/ml in complete media and put into 60 mm dishes (Greiner Cell Star, Stonehouse) pre-coated with Vitrogen-100 (Cohesion Technologies, Palo Alto, USA). The ATII cells were subsequently adhered at 37°C for 24 hours. The medium and any remaining contaminating cells were removed and fresh complete medium added. The cells were incubated for 16 hours, the medium removed and the cells washed with HBSS. Fresh complete medium was added and the cells incubated for a further 24 hours to establish confluent monolayers with ATII cell morphology confirmed as previously described[24]. ATII cell phenotype was confirmed by positive staining for alkaline phosphatase and mRNA transcripts for SP-C and aquaporin-3. Morphological characteristics were confirmed by electron microscopy. ATI cell phenotype was excluded by negative staining for aquaporin-5.

Purified cells were then either cultured with the pro-inflammatory agents LPS (10 μg/ml), TNF-α (10 ng/ml), IL-1b (1 ng/ml) for 4 hours prior to RNA extraction and/or sequential cell culture supernatant sampling or used in the proliferation assays described below. A minimum of 10 subject samples were used for each experiment. In vitro these cells rapidly differentiate and this precludes a prolonged culture timepoint.

### VEGF isoform-specific RT-PCR

Human and murine RNA was extracted using TRIzol reagent. Reverse transcription (RT) was carried out using the Superscript II system (Invitrogen). Beta_2_-microglobulin (B_2_M) was used as a housekeeping gene. Amplifications were carried out in a 20 μl reaction volume containing 13 μl RNase free water, 1.2 μl 25 mM MgCl_2 _(final concentration 1.5 mM) (Abgene), 0.4 μl 25 μM dNTPs (Abgene), 1 μl of 20 μM forward and reverse primers, 2 μl 10× reaction buffer (Abgene) and 0.4 μl 5 U/μl Taq DNA polymerase (Abgene). The following PCR conditions were used: denaturation at 94°C for 3 minutes, annealing at 72°C for 30 seconds then denaturation at 94°C for 30 seconds. The coding sequences for human VEGF (accession no. NM_000493, NM_001844, and NM_000088, respectively) and murine VEGF (MN_00441242 for mouse VEGF) were used to design primers using the online software, Primer3 (Whitehead Institute for Biomedical Research, Cambridge, MA). The primers span intronic junctions to avoid the amplification of genomic sequences. Primer sequences used were as in Table 1. Amplified products were visualized by gel electrophoresis using ethidium bromide (Sigma, Poole, UK) on a transilluminator (BioRad, Hertfordshire, UK) using semi-quantitative densitometric analysis (Biorad Geldoc software). Control reactions were run without the addition of reverse transcriptase. Previous experiments had determined the RT-PCR data would be in linear phase at a cycle number of 35 indicating the data were truly semi-quantitative. The inter-assay variability of densitometry measurements was 11.2%.

**Table 1:** Table of primer sequences for semi-quantitative RT-PCR for human VEGF and beta-microglobulin (B_2_M)

**RT-PCR Product**	**sense**	**antisense**
human VEGF_121,165,189_	5'-GAGATGAGCT TCCTACAGCAC-3'	5'-TCACCGCCTCGGCTTGTCACAT-3'

B_2_M	5'-GCATCATGGAG GTTTGAAGATG-3	5'-TAAGTTGCCAG CCCTCCTAGAG-3'

### Quantitative real-time RT-PCR

Quantitative real-time RT-PCR for VEGF isoforms was performed in a 25 μl reaction volume containing 12.5 μl of the SYBR Green PCR master mix (Sigma, Poole, UK), 5 μl of the RT reaction mixture, and 300 nM each primer using the Smart Cycler II System (Cepheid, Sunnyvale, CA). A standard curve was generated for each test mRNA using 8 serial dilutions from neat RNA to 1:200,000. The amplification program consisted of initial denaturation at 95°C for 2 minutes followed by 40 cycles of 95°C for 15 seconds, annealing at 58°C for 30 seconds, and extension at 72°C for 15 seconds. Primer sequences were as in Table 2. Commercially available GAPDH primers were used  and used as a reference for normalization in all RT-polymerase chain reactions (RT-PCRs).

**Table 2:** Table of primer sequences for quantitative real time PCR (QPCR) for human and murine VEGF

**QPCR Product**	**sense**	**antisense**
human VEGF_165_	5'-ATCTTCAAGCCATCCTGTGTGC-3'	5'-CAAGGCCCACAGGGATTTTC-3'

human VEGF_189_	5'-ATCTTCAAGCCATCCTGTGTGC-3'	5'-CACAGGGAACGCTCCAGGAC-3'

murine VEGF_120_	5'-AACGATGAAGCCCTGGAGTG-3'	5'-TGAGAGGTCTGGTTCCCGA-3'

murine VEGF_164_	5'-AACGATGAAGCCCTGGAGTG-3'	5'-GACAAACAAATGCTTTCTCCG-3'

murine VEGF_188_	5'-AACGATGAAGCCCTGGAGTG-3'	5'-AACAAGGCTCACAGTGAACG-3'

### VEGF ELISA

Cell culture supernatants were assayed for VEGF (VEGF_121_, VEGF_165 _only) using a commercial ELISA kit (R&D Systems). In brief, a specific monoclonal antibody was coated onto a microplate. Standards and samples were added and a polyclonal detection antibody added. The resultant colour developed in proportion to the amount of growth factor present and was read spectrophotometrically. The detection limit was 3 pg/ml.

### Cell Proliferation Assay (^3^H-thymidine incorporation)

ATII cells were seeded in 24-well plates (Greiner bio-one Ltd, Stonehouse, UK) in complete medium (10%NCS/DCCM/1% penicillin/streptomycin/amphotericin B) (Sigma, Poole, UK) at 100,000 cells per well. For 48 hours the cells were incubated in 5 ng/ml VEGF_165 _± 10 ng/ml *sflt*, 10 ng/ml KGF (as a positive control) and 10 ng/ml *sflt *(as a specific VEGF inhibitor) alone. The concentrations of KGF and *sflt *correspond to previously published concentrations in primary cell studies. 5 ng/ml VEGF_165 _approximates to epithelial lining fluid concentrations of VEGF in normal subjects and also following recovery from ARDS[4,8]. The cells were then washed with HBSS (Sigma, Poole, UK) and incubated in complete medium. Recombinant proteins and 37 kBq methyl-[^3^H] thymidine (Amersham Biosciences) were added to each well. At 48 hours incubation at 37°C, the cells were washed with trichloracetic acid 5% in PBS and then solubilized by adding 0.5 ml of 0.3 M NaOH (Sigma, Poole, UK). Cell lysates were subsequently pipetted into scintillation vials (Fisher Scientific UK Ltd, Loughborough, Leicestershire, UK) containing 2 mls of scintillation liquid (Amersham Biosciences) and counted by a β-counter; Beckmann Instruments (Beckman Coulter Ltd, High Wycombe, Buckinghamshire, UK).

### Cell count

ATII were seeded in 24-well plates (Greiner Bio-one Ltd, Stonehouse, UK) in complete medium, at 100,000 cells per well. After 48 hours, cells were washed with HBSSS and the protein of interest added. After 48 hours incubation, cells were washed with PBS and incubated with 100 μl of Trypsin-EDTA (Sigma, Poole, UK). When the cells were unattached, 100 μl of culture medium containing 10% FBS was added. Cells were then counted under the microscope using a haemocytometer.

### Statistics

Data were analyzed by ANOVA with Bonferroni *post ho*c multiple comparison correction using GraphPad Prism version 4.0 software. A p value of < 0.05 was considered significant.

## Results

### Human VEGF lung expression and quantification in normal, early and late ARDS

**Figure 1:**
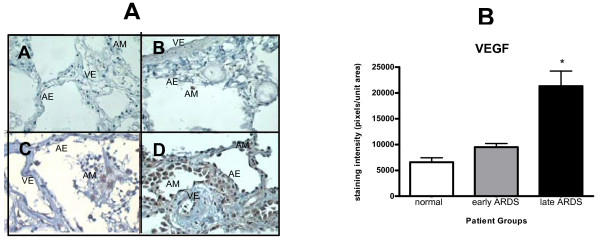
**A) Immunocytochemistry of human whole lung tissue (× 40) showing (A) isotypic control (from later ARDS), (B) normal controls, (C) early ARDS, (D) later ARDS (magnification × 40)**. Immunostaining shows positive VEGF expression in alveolar epithelium (AE), alveolar macrophages (AM) and (to a lesser extent) vascular endothelium (VE). Significant increase in staining noted in ARDS, especially later ARDS. Staining was assessed semiquantitatively using Histometrix software analysis. B) Histometric analysis of human VEGF in healthy human lung, early and later ARDS. *P < 0.001 in later ARDS versus early ARDS and normal, (ANOVA with post-hoc Bonferroni). Data are normal and plotted as mean and standard error.

Human VEGF expression was noted on alveolar epithelium and macrophages with weaker endothelial expression in all human tissue sections. VEGF expression was significantly increased in late ARDS compared to both normal subjects and early ARDS (p < 0.001) (Figure 1). This represented all VEGF isoforms both soluble and membrane-bound.

### Human VEGF isoform RT-PCR in normal, early and late ARDS

**Figure 2:**
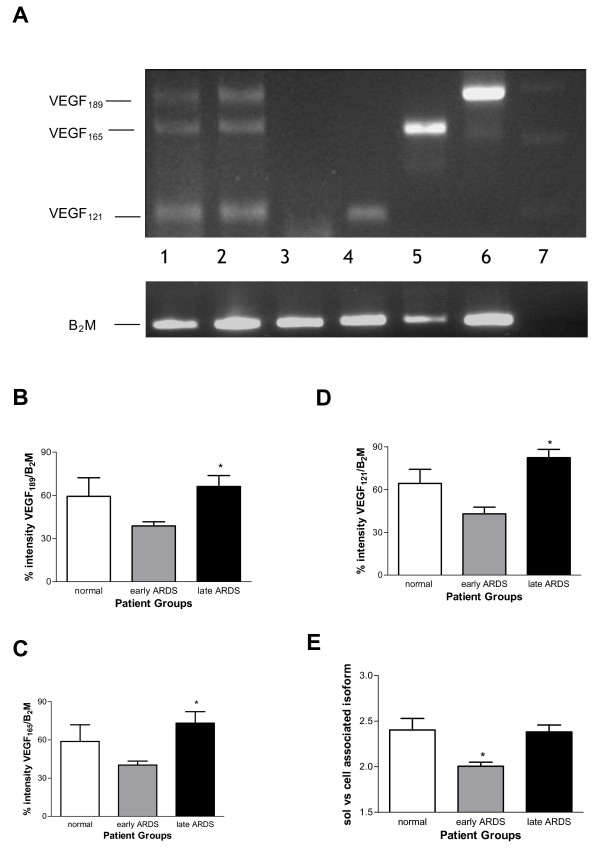
**A): Representative RT-PCR gel for human VEGF isoforms in normal, early and later ARDS, lanes as follows: (1) early ARDS, (2) later ARDS, (3) negative control, (4,5,6) sequenced positive controls (VEGF_121_, VEGF_165 _and VEGF_189 _respectively), (7) 100 kb ladder (bottom marker denotes 100 bp, top marker 300 bp)**. B-E): Semiquantitative densitometry of (B) VEGF_189_, (C) VEGF_165_, (D) VEGF_121 _relative to β-microglobulin (B_2_M) in normal, early and later ARDS (*p < 0.05 late versus early ARDS), (E) ratio of soluble (VEGF_121_, VEGF_165_) to cell-associated (VEGF_189_) isoforms (*p < 0.05 early versus normal and later ARDS). Data are plotted as means with bars denoting standard errors (ANOVA with post-hoc Bonferroni) for B-E).

In order to determine the individual VEGF isoforms, RT-PCR was initially used. A representative RT-PCR gel from the human FFPE tissue is shown in Figure 2a. When individual isoforms were compared between groups, a significant increase in all three isoforms was detected in later versus early ARDS (p < 0.05) as seen in Figure 2b–d. However, when considering individual relative isoform production in disease there was a significant decrease in the relative ratio of soluble to cell-associated isoforms in early ARDS (p < 0.05) as seen in Figure 2e (comparing combined densitometry data).

### Murine VEGF mRNA expression

**Figure 3:**
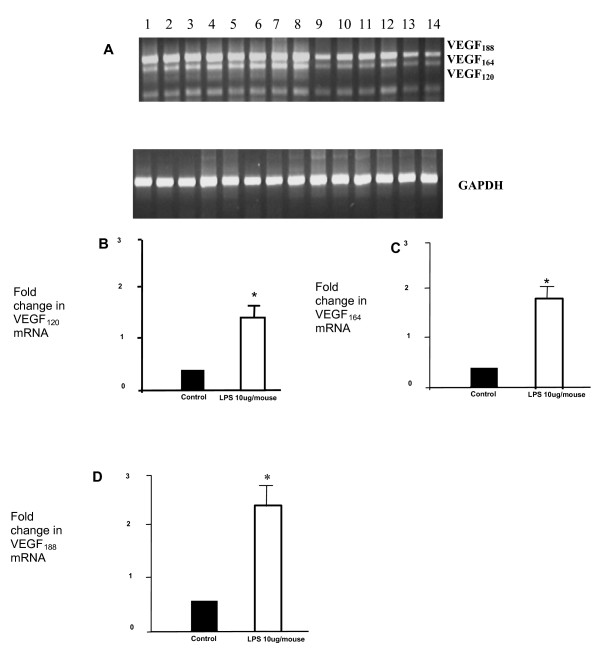
**4 samples were examined for each treatment group**. A) Representative RT-PCR gel of VEGF isoforms in control and injured murine lung. Lanes are as follows: (1–8) injured murine lung at day 5 (24 hours post last LPS dose) (9–12) injured murine lung at day 2 (24 hours post last LPS dose), (13–14) control murine lung. B-D): Further real-time PCR analysis was undertaken in the samples at day 5. This confirmed the increased levels of VEGF_120_, VEGF_164 _and VEGF_188 _in injured murine lung (*p < 0.05 fold change compared to control).

The human samples used were of archival post-mortem origin and hence potentially subject to post mortem degradation although a recent study suggests that this is minimal[25]. In order to support these human findings, we repeated the mRNA analysis in samples extracted from snap frozen murine whole lung injury samples. We demonstrated a significant fold increase in all isoforms (p < 0.05) (Figure 3). Analysis of absolute values showed a significantly greater amount of VEGF_188 _than VEGF_164 _and VEGF_120 _(p < 0.05).

### ATII VEGF mRNA isoform expression (RT-PCR)

**Figure 4:**
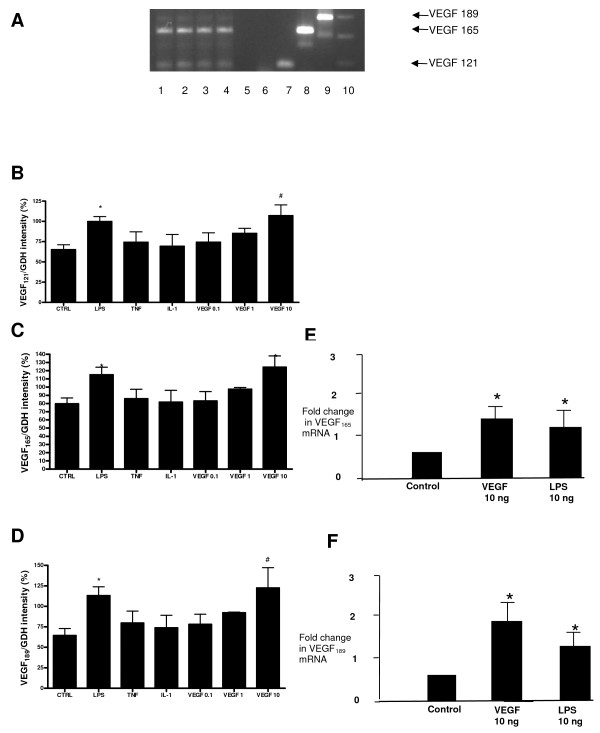
**A) Representative RT-PCR gel of VEGF isoforms in ATII cells**. Lanes are as follows: (1–4) human ATII cell samples, (5) blank, (6) negative control, (7–9) sequenced positive controls for VEGF_121_, VEGF_165 _and VEGF_189 _respectively, (10) 100 kb ladder (bottom marker denotes 100 bp, top marker denotes 300 bp). B-D): Semiquantitative densitometry data (n = 8, in triplicate) showing VEGF_121_, VEGF_165_and VEGF_189 _in response to LPS (10 μg/ml), TNF-α (10 ng/ml), IL-1β, VEGF_165 _(0.1,1,10 ng/ml) at 4 hours. Data are plotted as mean with bars denoting standard error. *P < 0.05 (ANOVA post hoc Bonferroni) 10 μg/ml LPS versus control (all isoforms) and 10 ng/ml VEGF_165 _versus control (VEGF_165 _only). ^#^P < 0.01 (ANOVA, post hoc Bonferroni) 10 ng/ml VEGF versus control (VEGF_121 _and VEGF_189_). E-F): Further real-time PCR analysis confirmed the effect of LPS and VEGF on VEGF_165 _and VEGF_189 _(*p < 0.05 fold change compared to control).

The main source of VEGF on the basis of our immunocytochemistry results were ATII cells so we isolated these for further investigation. We found that ATII cells constitutively express mRNA for all the three main VEGF isoforms: VEGF_121_, VEGF_165 _and VEGF_189 _(Figure 4a). All isoforms were significantly increased in comparison to control following treatment with 10 ng/ml VEGF_165 _(p < 0.05 for VEGF_165_, p < 0.01 for other isoforms) and 10 μg/ml LPS (p < 0.05) at 4 hours (Fig 4b–d). Other pro-inflammatory stimuli (TNF, IL-1) and lower concentrations of VEGF_165 _did not alter relative VEGF isoform expression. These results were confirmed by Q-PCR showing a doubling of VEGF_189 _and VEGF_165 _compared to control (p < 0.05) in response to LPS 10 μg/ml and VEGF 10 ng/ml (Figure 4e–f).

### ATII VEGF protein levels and response to LPS

**Figure 5:**
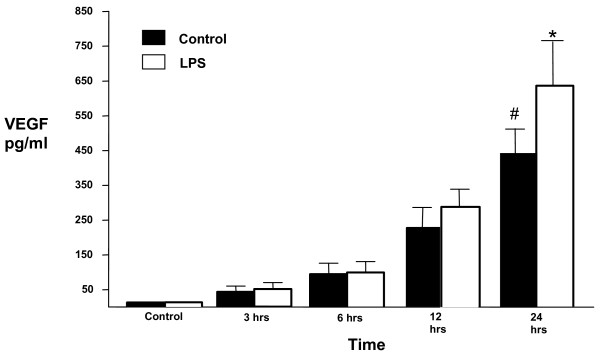
ATII cell supernatant levels of VEGF protein significantly increases with time in unstimulated cells, unstimulated cell supernatants control v 24 hrs, filled bars, (#p < 0.01) with a significant increase in response to LPS 100 ng/ml at 24 hours, unfilled bars, (*p < 0.05), ANOVA with post hoc Bonferroni.

Having determined the production of VEGF isoforms at the mRNA level we went on to assess the release of the soluble isoforms (VEGF_121_, VEGF_165_) by the cultured cells. ATII cells express significant amounts of VEGF_121 _and VEGF_165 _constitutively (Figure 5). These levels significantly increased with time in human ATII supernatant (p < 0.01 vs control at 24 hours). At 24 hours, LPS (100 ng/ml) further stimulated VEGF production (p < 0.05).

### ATII proliferation

**Figure 6:**
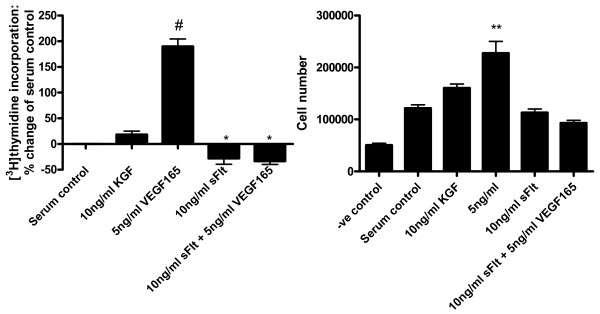
**Bar graphs depicting ^3^H-thymidine incorporation into ATII cells (A) and cell number (B) following treatment for 48 hours with VEGF 5 ng/ml, *sflt *10 ng/ml and combination confirming a significant proliferation with VEGF 5 ng/ml (#p < 0.001 vs serum control)**. The presence of *sflt *not only inhibited the proliferative effect of VEGF but also reduced proliferation below that of serum controls (*p < 0.05), suggesting an autocrine effect. Cell number also increased significantly with 5 ng/ml VEGF_165 _(**p < 0.01) compared to serum control. The addition of *sflt *alone and in combination with VEGF_165 _showed no significant difference compared to serum control (p > 0.05) but did significantly inhibit VEGF_165_-induced proliferation (p < 0.001 compared to 5 ng/ml VEGF_165_).

Having determined the production of VEGF by the ATII cell population, we explored the potential effect of VEGF_165 _on these cells. An increase in human ATII cell proliferation as assessed by ^3^H-thymidine was detected with 5 ng/ml VEGF_165 _(p < 0.001), comparable to levels detected previously in bronchoalveolar lavage fluid[8]). Furthermore, with the addition of a natural VEGF inhibitor, soluble VEGFR1 (*sflt*), there was a significant reduction in proliferation compared to serum control (p < 0.05) suggesting an autocrine effect (Figure 6). The addition of *sflt *alone and in combination with VEGF_165 _showed no significant difference in cell number compared to serum control (p > 0.05) but did significantly inhibit VEGF_165_-induced increase in cell number (p < 0.001 compared to 5 ng/ml VEGF_165_). This suggested that VEGF_165 _was inducing proliferation rather than survival alone.

## Discussion

Previous observational data by ourselves and others have demonstrated plasma VEGF levels rise and intrapulmonary levels fall in the early stages of lung injury with normalization of both during recovery[7,8]. Similarly, reduced levels of VEGF have been described in normal smokers and patients with idiopathic pulmonary fibrosis (IPF); other conditions in which damage to the alveolar epithelium may be present[26].

Potential explanations for the apparent reduction in intrapulmonary VEGF levels in early ARDS are manifold and not mutually exclusive. They include increased membrane-bound rather than soluble isoforms, changes in isoform expression and damage to the alveolar-capillary membrane with consequent leakage of intrapulmonary VEGF into the vascular bed [27-30].

We therefore assessed expression of VEGF and its specific isoforms by immunohistochemistry and isoform-specific RT-PCR (as isoform-specific antibodies are not available) in archival normal and ARDS lung tissue. We demonstrated a significant up-regulation of VEGF in later ARDS tissue compared to normal subjects. However in early ARDS, in contrast to our epithelial lining fluid (ELF) findings we did not detect a reduction in total VEGF expression[8]. One other group has also explored this area, but using different timepoint characteristics, whole tissue homogenates including inflammatory cells and did not assess differential isoform expression[11]. In both these studies ELISA methodology was used which detects only the soluble isoforms, VEGF_121 _and VEGF_165_[8].

In order to consider changes in isoform expression as a possible explanation for these discrepancies, we assessed mRNA as there are no isoform-specific antibodies currently. No significant reduction in VEGF isoform expression occurred in early ARDS in comparison to normal subjects although a trend was suggested. However, significant changes were detected between early and later ARDS in all isoforms and there was a significant decrease in the relative ratio of soluble to cell-associated isoforms in early ARDS compared to later ARDS and normal subjects. In the context of our previous ELF findings, this data is supportive of the suggestion that isoform switching is a critical regulatory mechanism for VEGF bioactivity in that isoform-specificity may be important in ligand-VEGF receptor interactions[31]. Furthermore, there is more potential for soluble VEGF to have extrapulmonary effects which is extremely relevant since the most common cause of death in ARDS is multi-organ system failure[32,33].

Obtaining ARDS lung tissue is limited by the lack of surgical biopsies of this disease in our clinical practice and theoretically necropsy lung tissue might introduce selection bias for a more severe spectrum of ARDS, as intrapulmonary VEGF levels are known to be lower in non-survivors with ARDS[8]. There was no evidence of any significant lung disease in the normal necropsy lung tissue, but it is conceivable that the extra-pulmonary disease process contributing to death might have affected VEGF levels although recent data suggests this is not the case[25]. In order to investigate this possibility we repeated this analysis in snap frozen lung tissue from our multiple dose LPS-induced lung injury model at day 5 post initial injury, reflecting early ARDS [23]. These data supported the human post mortem findings of an increase in cell-associated VEGF in an ARDS situation.

Previous *in vitro *studies had confirmed VEGF is abundant in lung and suggested the alveolar epithelium as a key source [5,6,34]. Several lines of *in vitro *evidence have pointed to a possible role for VEGF in lung repair and recovery following injury[19,29,35,36]. In one LPS-induced murine model of lung injury, intrapulmonary levels of VEGF increased following injury for 96 hours, mirroring the increase in bronchoalveolar lavage fluid protein and neutrophils with significant VEGF localization to lung epithelium but increases mainly in inflammatory cells [37]. However, previous experiments performed in our laboratory have confirmed pg/ml levels of expression of VEGF in cultured alveolar macrophage supernatants from patients with ARDS and "at risk" of ARDS suggesting that they are unlikely to be the main cellular source of VEGF, although they may contribute [8]. In both newborn and adult rabbit, hyperoxic lung injury resulted in a relative reduction in VEGF_189 _and parallel increase in VEGF_121 _and VEGF_165 _mRNA expression with normalization to control values during recovery[38]. Therefore, we went on to investigate the role of human ATII cells as both a source and potential target for VEGF bioactivity.

We initially established at the mRNA level that ATII cells express the major VEGF isoforms (VEGF_121,165_) (both soluble) and VEGF_189 _(membrane-associated). The specific functions of these isoforms have not been clearly identified in humans although genetically modified mouse models suggest they may be significant. Ideally, we would have isolated ATII cells from ARDS lung biopsies and undertaken mRNA analysis but this was not possible as described above. We have also shown that VEGF_165 _is differentially upregulated by various exogenous compounds, in particular LPS.

We demonstrated for the first time that primary human ATII cells constitutively produce soluble VEGF (VEGF_121,165_) isoforms in a dose-dependent manner that increased in response to LPS (p < 0.05). This is in agreement with other studies showing high ELF levels in normal human subjects[4]. The increase in constitutional production in time would suggest active secretion. The relationship of these findings at the protein level to those at the mRNA level is not clear-cut. The ELISA used does not differentiate between VEGF_121 _and VEGF_165 _and only detects the unbound free protein. Cell-associated VEGF is not detected and may be considerable.

The high intrapulmonary levels of VEGF and its changes in ARDS led us to hypothesise that VEGF may be an epithelial mitogen or survival factor. This has particular relevance in repair following injury as occurs in ARDS when the alveolar epithelial surface must be regenerated to clear fluid and restore the normal ATI cells and gas exchange[39]. The evidence for VEGF as an epithelial mitogen conflicts. Proliferation in human fetal explants[18] and acid-injured A549 cells[19,40] and surfactant production by murine ATII cells[36] have been described, but these data were not supported when rat ATII cells were used[20]. In the current study, we have shown for the first time that VEGF at 5 ng/ml (akin to normal human ELF VEGF levels), induces significant proliferation of these cells which is inhibited by the specific inhibitor *sflt*. Furthermore, the reduction in proliferation by the addition of *sflt *suggests a potential autocrine effect of this protein.

We have only explored the effects of VEGF_165 _in this study and other isoforms may also be relevant. In addition, sample numbers are limited and subject to biological heterogeneity inherent in primary cell studies and our preliminary findings need to be expanded upon and analysed individually in greater depth using techniques such as laser capture microdissection for single cell PCR analysis. VEGF_189 _can be cleaved in into smaller units. Therefore, repeating these experiments with a cleavage inhibitor should be considered in the future. The relevance of this is that the concept of soluble versus cell-associated isoforms has not yet been fully resolved and proteolytic cleavage may alter isoform ratios in ways not detected in this study.

In conclusion, we present evidence that changes in VEGF isoforms occur between early and late ARDS. These data are supported by both murine model and isolated human ATII cell data. We have demonstrated ATII cells to be a source of VEGF isoforms upregulated by lipopolysaccharide, often implicated in the ARDS process. Finally we show evidence that VEGF_165 _is an ATII cell mitogen, inducing proliferation which was inhibited by soluble VEGFR1. These data suggest a key role for VEGF bioactivity in lung injury and ARDS.

### Ethics approval

The protocol was approved by the North Bristol NHS Trust Local Research Ethics Committee.

## Competing interests

The authors declare that they have no competing interests.

## Authors' contributions

ARLM carried out the immunohistochemistry, FFPE RNA extraction, ATII cell isolation and culture, ELISA, semiquantitative RT-PCR, statistical analysis, drafted the manuscript and contributed to its design and conception. SKD carried out the proliferation studies and drafting of part of the manuscript. SIHG designed and generated the murine model and performed the murine RT-PCR. KMU and LA contributed to the ATII cell isolation and culture. KMG and BZ performed the real time PCR and contributed to drafting of part of the manuscript. TDT contributed to the ATII cell culture and drafting of part of the manuscript. NBN contributed to the immunohistochemistry and drafting of part of the manuscript. ABM conceived of the study, contributed to its design and drafted the manuscript. All authors read and approved the final manuscript.
